# Re-thinking Women's Sport Research: Looking in the Mirror and Reflecting Forward

**DOI:** 10.3389/fspor.2021.746441

**Published:** 2021-10-11

**Authors:** Katie Lebel, Ceyda Mumcu, Ann Pegoraro, Nicole M. LaVoi, Nancy Lough, Dunja Antunovic

**Affiliations:** ^1^Ted Rogers School of Management, Ryerson University, Toronto, ON, Canada; ^2^Pompea College of Business, University of New Haven, West Haven, CT, United States; ^3^Lang School of Business and Economics, University of Guelph, Guelph, ON, Canada; ^4^School of Kinesiology, University of Minnesota, Minneapolis, MN, United States; ^5^College of Education, University of Nevada, Las Vegas, NV, United States

**Keywords:** women's sport, women athletes, professional sport, sport industry, sport strategy

## Abstract

Despite decades of research and advocacy—women's professional sports continue to be considered second class to men's sports. The goal of this paper is to rethink how we state, present, and solve problems in women's sport. To affect true change, the wisdom of a broad stakeholder group was embraced such that varied perspectives could be considered. A three-question survey was developed to examine what key constituents believe is working in women's sports, what they believe the salient challenges are for women's sport, and how they would prioritize the next steps forward in the post-pandemic sport landscape. Results indicated siloed differences of opinion based upon the age and role of the stakeholder in the women's sport ecosystem. We discuss the implications and offer recommendations as to how we as scholars might recalibrate our approach to women's sport scholarship to maximize the impact of our research and affect change.

## Introduction

While women's sport research has undoubtedly progressed, only a few token statistics have broken through into mainstream conversation and informed industry decision-making over the past decade. Arguably the most popular academic reference in women's sport refers to research that has tracked media coverage inequities. The oft-cited 4% statistic refers to the percentage of coverage afforded to women's sport in sports media. It is regularly used to provide context in industry conversations and simultaneously exists as the most viewed, downloaded, and cited statistic in the *Communication and Sport* academic journal (Cooky et al., 2015). Why have so few other scholarly contributions been able to achieve this kind of reach? While we recognize that research alone will not create change (Fink, 2015), as we reflect, reset our collective agendas and begin to build back and step forward in the post-pandemic sport landscape, rethinking our research contributions to more closely align with key stakeholders could help us to better serve both women athletes and the growth of women's professional sports.

Scholars have commonly shared and challenged the same linear storylines about women's sport for decades—comparing women's sports to men's sports, relating pre-Title IX to current day, highlighting the gendered participation gap and resource disparities, noting the decline and stagnation of women in sport leadership positions, documenting the dismal and marginalized media coverage, and detailing a perceived lack of fan interest (e.g., Hardin, 2005; Schultz, 2014; Burton, 2015; Bruce, 2016; LaVoi, 2016). Despite our best intentions to educate and inform around gender equity, in practice, we've witnessed little perceptible change. Amidst a time of rapid disruption and innovation, perhaps it is time to also reframe the ways in which we collectively study, think, and talk about women's sport.

Wedell-Wedellsborg (2020) suggested the process of reframing can be a helpful strategy to solve difficult, complex problems. His framework encourages problem solvers to look outside of traditional frames, re-think their goals, examine bright spots, and then look in the mirror to let go of past assumptions and narratives, which ensures outside perspectives are taken into account. The goal of this framework is to maintain momentum and move forward. As we consider our academic expertise, it's critical to remember that we tend to frame problems that match our preferred solutions (Wedell-Wedellsborg, 2020). However, are there stakeholders whose influence and insights we might be missing? Are we overlooking systemic factors that might influence different stakeholder groups in ways we haven't conceived? What are we not paying attention to? How is functional fixedness—a type of cognitive bias that involves a tendency to see objects as only working in a particular way—affecting our research?

Grant (2021) noted recently the art of reconsidering has never been more critical. Accelerated change is evidenced in a number of fields by the global COVID-19 pandemic, causing us all to doubt previous practices and re-imagine new possibilities. While momentum for gender equity in sport was building prior to COVID-19 (Leberman et al., 2020; Bowes et al., 2021), the forced pause necessitated by the pandemic inarguably served as a pivot point for women's sports, showcasing its great potential for growth. We now find women's sports in a moment of transition. The sports industry is actively re-thinking how women's sports are produced, measured, and distributed (Sport Innovation Lab, 2021). As thought leaders in academia, we too must grapple with the idea that facts and the context may have changed over the past decade and recognize the potential value of reframing our own minds (Grant, 2021).

Our mission with this research is to cultivate curiosity, challenge assumptions, question the status quo, and promote an innovative research culture that can help to move the *entire* field of women's sport forward by driving strategy and building theory. We want to pause and rethink how we state, present, and solve problems in women's sport so that we are better positioned to affect sustained and real change. In line with Wedell-Wedellsborg's problem-solving framework, the first step in this re-framing journey is to embrace the wisdom of crowds. The purpose of this research, therefore, was to actively engage a wide variety of stakeholders in the women's sport ecosystem (i.e., all those with an interest in women's sports) to better understand the state of the women's sport landscape from a variety of perspectives and determine whether or not differences exist between stakeholder groups.

## Literature Review

The following review addresses the key elements within Wedell-Wedellsborg's problem-solving framework. We begin by prefacing how we as academic researchers might begin to look outside of our traditional frames and rethink our research goals. We then look to the “bright spots” in women's sport research, summarizing the types of research that are working well. This is followed by a critical “look in the mirror” where we consider potential blind spots in our research and examine academic bias in our field. We conclude with a preliminary reflection on the variety of stakeholders in the women's sport space and the diversity of problems each may be trying to solve as we look forward.

### Looking Outside the Frame

What are we missing? Solving complex problems requires us all to get curious about what we do not know, let go of past ways, dated assumptions, and familiar narratives. We all need to admit that “we don't know what we don't know,” and trust that other women's sport stakeholders could bring value to the reframing process with new perspectives and insights. Nutt (2002) noted that one of the most powerful things one can do when solving problems is generate multiple opinions to inform the ideation process (Nutt, 2002). Wedell-Wedellsborg (2020) built on this and promoted inviting outsiders to come up with alternative framings, suggesting that the strategy of zooming out to ask others what's missing can be a powerful way of bringing about a more people-oriented lens to a problem. This approach helps us to look beyond our traditional framing tendencies and/or see old problems in new ways. It also has the potential to be particularly effective as it relates to the industry-academic chasm in women's professional sport which has traditionally fractured around industry desire to monetize research findings on an efficient timeline and academic interests that value quality, rigor, and theoretical development for long-term sustainability. Wedell-Wedellsborg (2020) highlights Kaplan's Law of the Instrument, noting that while it's not necessarily a bad thing to have a default solution, if you've repeatedly failed to solve a problem with your preferred solution, there's a good chance you need to reframe the problem. Specific to the challenges of women's sport, we recognize that real change will require multiple perspectives—from industry and academia, across a range of stakeholders, ages, backgrounds and identities—coming together to challenge one another and move women's sport forward into the future.

### Examine Bright Spots

What type of research piques industry interest or impacts practical change? As we move to “look outside” of our academic frame, Wedell-Wedellsborg encourages us to look for “bright spots” to help gain new perspectives (p. 84). Grant (2021) similarly suggests the creation of “a specific kind of accountability—one that leads people to think again about best practices” (p. 216). So, what in women's sport research is working well? As noted, the media representation work of Cooky et al. (2015) and the most recent iteration of the longitudinal study, Cooky et al. (2021), have become leading industry references. What sets this work apart? The research offers “sticky stats” that can be easily distilled for industry amplification while also serving to effectively quantify the vast inequities in women's sport media. Sticky stats are typically publicly available (as opposed to being behind a paywall) and they can be easily visualized and shared, which means they can simplify the translation of academic work and increase reach and traction. What's more, sticky stats are often startling.

The sticky stat strategy is being used more frequently in both industry reports and media reporting around women's sports. For example, the commercial investment audits conducted by the Women's Sport and Fitness Foundation popularly revealed that women's sport sponsorship accounted for just 0.4% of total sports sponsorships between 2011 and 2013 (WSFF, 2014). Ernst and Young's sticky stat that 94% of women in C-suite offices played sport has also been widely shared (EY, 2015). Deloitte made headlines with their forecast that women's sports were on track to become a billion-dollar industry (Lee et al., 2020). The longitudinal report cards that track the progress of women sport coaches and leaders (i.e., the Tucker Center for Research on Girls and Women's annual *Women in College Coaching Report Card* or The Institute for Diversity and Ethics in Sport Diversity Reports) are also widely cited data points and have become well-regarded industry sources.

In the media, sticky stats have started to make for great headlines. Journalists seem to be drawn to flashy viewership numbers, social media engagement records and social activism. For example, several outlets reported on the fact that the WNBA's viewership was up 74% after just five games in the 2021 season (Carp, 2021). It was also widely reported that NWSL teams received a league-high 12 million Twitter impressions during the 2021 Challenge Cup tournament (Brennan, 2021), and Naomi Osaka's tweet focused on engaging conversation around mental health ahead of the French Open was not only lauded around the world, it was also noted for generating more than 41 million impressions across Twitter and Instagram (Irelan, 2021).

The concept of data journalism or data-driven journalism has also become a popular means to elevate journalistic storytelling (Boyle, 2017). In the case of women's sport, data are a wonderful tool to educate audiences. Letting the data tell the story is an effective way to push back against the deeply-engrained opinions and stereotypes that have plagued the women's sport space. This practice is being successfully employed in women's sports by journalists on untraditional platforms like Lindsay Gibbs' *PowerPlays* newsletter, *The Black Sportswoman Newsletter*, or the social media news source, *Shot:Clocks Media*. We could also look to the example of new media platforms like *The Gist* or *Just Women's Sports*, both of which recently received millions of dollars in seed funding for their roles in filling the void in the women's sport media marketplace. If one of our goals as advocates for women's sport is to broaden the reach of our work, infusing these journalistic styles into the reporting of our research through accessible media (e.g., opinion editorials, podcasts, infographics), or even collaborating with these noted outlets to help support their research needs could be effective ways for academics to re-think our knowledge translation plans and educate a broader population through public scholarship (Yapa, 2006; Colbeck and Weaver, 2008).

Information does not simply manifest itself into public discourse. Colbeck and Weaver (2008) encouraged faculty to see, explore, and exploit the connections between public scholarship and other faculty responsibilities including research, teaching, and service, noting it is “an important way to leverage public scholarship engagement” (p. 28). Yapa (2006) advocated for intentionality in academic research, arguing “new knowledge is created in its application to the field” (p. 73). To this end, we examine what Wedell-Wedellsborg (2020) refers to as a “positive outlier.” In lieu of the traditional mode of publishing an academic paper and *then* promoting work in media outlets, Isard and Melton (2021) recently teased the results of their research on the media coverage of Black players in the WNBA in a *Sports Business Journal* OpEd. They discussed a topline finding—that Black WNBA players receive significantly less media coverage—and were immediately afforded a series of high-profile press opportunities after the piece was published. The research was praised by ESPN employees and Paige Bueckers, a top collegiate basketball player, referenced the research in her nationally televised acceptance speech upon winning an ESPY for college athlete of the year, further amplifying the research. In this example, the reconsideration of traditional academic processes allowed the research findings to be reframed for multiple audiences, vastly extending both its reach and impact.

It's easy to fall victim to negativity bias when working to solve problems (Wedell-Wedellsborg, 2020). In studying the bright spots of women's sport research, we enjoy an opportunity to pay more attention to situations in which things went particularly well. Perhaps unconventional by academic standards, Isard and Melton's decision to re-think the traditional order of knowledge transfer in academia allowed the researchers to capitalize on a moment in women's sport and embrace an opportunity to educate the industry. Had they waited to publicly unveil their work until the typically slow academic publication process was complete, would the same opportunities have existed? Could this strategy be successfully replicated with future research? Perhaps paying attention to the behaviors and circumstances that surround bright spots, could lead us to rethink the ways in which we communicate our research.

### Look in the Mirror

What does academia bring to the table? What is our role in creating the problem? Wedell-Wedellsborg (2020) notes that when considering problems, we often overlook our own role in the situation. Collectively, both scholars who specifically research women's sport and those involved more generally in the sport management academy, offer a nuanced understanding of the sport landscape and provide a vast methodological toolkit that allows us to advance understanding and create new knowledge. If we are to look in the mirror, however, it's incumbent upon us to recognize the bias toward men's sports that exists in both the current body of sport-related academic literature and sport management curricula.

When it comes to research, Fink (2015) provided a thorough review highlighting the broad scope of research dedicated to the media coverage, marketing, and promotion of female athletes, and women's sports. Burton (2015) and LaVoi (2016) similarly conducted a multi-level examination of the range of scholarship documenting women in sport leadership and coaching respectively. Bruce (2016) synthesized four decades of research on media representations of women's sports. Together, these reviews underscore the progress in scholarship dedicated specifically to women's sport. A recent study by Delia et al. (2021), however, noted that nearly all academic studies related to team identification have exclusively dealt with men's sport. Their review of hundreds of articles examining team identification as a focal variable found only five *articles* in which scholars explicitly studied the women's sport context (Delia et al., 2021). A review article of social media research in sport management featured 70 studies of which only two specifically focused on women's sports (Filo et al., 2015). These findings are not isolated examples in sport management literature, rather they are emblematic of the long-running assumption that men's and women's sport contexts are the same. The exclusion of the women's sport context in sport management research illustrates the extreme imbalance that exists across sport management literature and accentuates how we as scholars are “perpetuating the disparities between women's and men's sport” through our research agendas (Delia et al., 2021, p. 67).

Relative to sport management curricula, the women's sports context has to date been erased, ignored or omitted from course content in our field. Women are severely underrepresented in sport management programs with 40% of institutions offering a sport management degree reporting a female student population of 20% or less (Jones and Brooks, 2008). The perception of sport as a male dominated field gives the impression that an education in sport has limited options for women upon graduation (Tiel, 2002)^1^. Harris et al. (2014) examined female sport management students' perceptions toward their sport management degree and queried them about the sport management educational environment. Participants indicated experiencing negative feedback relative to their degree choice from parents, family members, friends, and college peers studying other majors. They also reported perceptions of a “chilly climate” in the program, detailing negative experiences that they believed were the result of their underrepresentation. We can and should do more to fix the gender imbalance within our own discipline. We must be more attuned to our internal and external self-awareness as academics and work toward overcoming our blind spots (Wedell-Wedellsborg, 2020).

### Take Their Perspective

What problems are other women's sport stakeholders trying to solve? Do we understand the variety of “crowds” we are trying to reach through our research? Wedell-Wedellsborg (2020) implores problem solvers to invest time and energy into learning how others see the world, and perhaps more importantly, understand how their views might differ from our own. Perspective-taking is cognitively complex. It involves understanding alternate contexts and worldviews (Wedell-Wedellsborg, 2020). In the case of women's professional sport, it also means understanding the current state of the women's sport landscape and recognizing the full variety of stakeholder perspectives that exist across the ecosystem. This is a key gap in our current understanding and in a rapidly changing sport environment, arguably the element of Wedell-Wedellsborg's framework where we have the most to learn.

If we are to effectively advance women's sport through scholarship, it is imperative that we understand the views and priorities of those we are trying to educate and protect against the pitfalls of academic groupthink. As such, the purpose of this research was to cultivate our understanding of women's sport stakeholders and explore their perspectives on the state of women's sport to determine both where differences might exist and where our energy as researchers might best be spent as we rethink the study of women's sport.

## Method

### Participants and Procedure

This study employed an exploratory, cross sectional design with purposive sampling aimed to collect insights from stakeholders in women's sport (i.e., those with an interest in women's sport) regarding the state of women's sports. Following IRB approval, the survey was broadly shared through a combination of academic and industry listservs and social media postings in October 2020. We encouraged participants to share the survey among their own networks to broaden and diversify our sample, while also attempting to eliminate potential bias by opening access to the survey beyond the listservs we accessed. No incentives were provided, and the survey remained open for one month. A total of 1,034 survey attempts were captured. The total sample of usable surveys for analysis was 383, as many surveys were incomplete. A largely recognized disadvantage of open-ended questions is the heavy burden on respondents (Dillman, 2007). Existing research suggests that while open-ended questions, can yield rich insights, they also have much higher rates of item non-response than other types of survey items (Miller and Lambert, 2014).

Respondents were predominantly from the US (71%) and Canada (14%), white (79%), and female (74%). The sample included representation from a variety of age groups and sport occupations, however, with 42% of the sample identifying as academic faculty, 47% identifying as industry practitioners, and 11% identifying as students. Within the industry practitioner group, respondents reported careers in advertising, corporate sales, promotions, ticket sales, community relations, media relations, player personnel, and medical and athletic training. A combined 8% of the industry practitioner group noted executive experience (e.g., CEO, COO, CMO, CFO). The sample also included coaches (5%), athletic administrators (4%), and sport practitioners currently out of work (4%). Table 1 includes a full description of respondent demographic characteristics.

**Table 1:** Demographic characteristics of respondents.

**Age**		**Gender**	
Gen Z	6.3%	Female	73.1%
Millennials	41.3%	Male	22.7%
Gen X	34.7%	Non-Binary	0.26%
Baby Boomers	10.4%	Prefer Not to Respond	3.7%
Prefer not to Respond	7.3%		
**Ethnicity**		**Occupation**	
Caucasian	78.6%	Academic Faculty	41.8%
African American/Black	5.9%	Industry Practitioner	26.3%
Latino/Hispanic	4.9%	Industry Executive	7.7%
Asian/Pacific Islander	2.9%	Student	11.2%
Two or More Races	3.4%	Coach	5.2%
Prefer Not to Respond	3.1%	Athletic Administrator	3.9%
		Currently Out of Work	3.9%

### Instrument and Analysis

A short survey was constructed in Qualtrics to collect insights on the state of women's sports with three questions posed: (1) What do you believe was working well in women's sport before COVID-19?, (2) What are the key challenges facing women's sport post COVID-19?; and (3) Going forward, what one change would you make that would provide the biggest impact for women's sport? In addition to the three open-ended questions, respondents' age, gender, ethnicity, country of residence and occupation were gathered. Respondents with missing data were removed from the dataset, and the remaining data were imported into Leximancer where a thematic analysis of the responses was conducted on the three open-ended questions. Comments were examined for each question separately by occupation group and age cohort, given these were the two variables with the greatest range.

Leximancer software was used to analyze the text responses to each of the questions. The software conducts a thematic analysis of written words (Bals et al., 2012). The analysis began with a few seed words and then continued to analyze large amounts of texts. The process generated a concept list (i.e., descriptors) that was statistically reliable and reproducible, as it was generated from the input text itself. This automated approach does not require investigation of coding reliability and validity (Angus et al., 2013) it is capable of analyzing vast sets of texts making it an effective tool for qualitative analysis (Bals et al., 2012), and it removes potential concerns around researcher bias. The output from Leximancer is a concept map or clustering of themes based on their relevance and connectivity within the data set. All content within each Leximancer-generated theme had the same context (see Frederick et al., 2016), and the relevance and connectivity of the themes provided the basis for identifying frames within the data. Once the themes were identified by the software, the researchers were able to dig deeper into the data to discover the nature of the dialogue within each theme and understand its nuance and context more accurately.

## Results

Upon reviewing the content of each theme and its relevance and connectivity to other themes, frames were formed within each of the three questions based upon occupation and age cohort. The meaning uncovered within each frame is discussed in the subsequent sections.

### Frames Within “What's Working”

The first question examined the perspectives of seven occupation groups on what they believed was working well in women's sports prior to the pandemic. Comments from these groups revealed two key frames among stakeholders: “*Growth of Women's Sports”* and “*Public Support for Women's Sport and Equity*.”

#### Growth of Women's Sports

“*Growth of Women's Sports”* emerged as a common frame within the comments of all occupation groups. However, respondent's perspectives of growth varied by occupation.

For athletic administrators and coaches, growth referred to increases in girls' and women's sport participation, the number of female coaches in college sports, a greater number of women athletes, and increased availability of women's sport programs. An athletic administrator noted “*making strides in equity both in playing conditions and remuneration, more opportunities for female student-athletes and better resources for women coaches*” as things that were working well, while a coach articulated that “*more opportunities were being created for female athletes, momentum was shifting toward hiring more females and coaches of color, and Title IX regulations [were helping administrators move forward] with an equal number of male and female sports at institutions*.”

Industry executives discussed the “*Growth of Women's Sports”* in terms of increased public awareness, corporate support, and a higher quality of media coverage. They also saw the athletic success of women athletes and the support of male allies as reasons driving this growth. One executive noted, “*sponsorship deals were starting to invest more than in the past*,” another highlighted that, “*within the last couple years, women's sports have had more exposure in the media in regards to televised events and having more support from men's major league sports and other organizations*.”

Respondents, who were out of work at the time of data collection, acknowledged increased coverage of women's sports on mainstream media during prime time and focused on increased visibility and awareness as indicators of growth. They also credited the COVID-19 pandemic for increased, higher quality media coverage of women's sports due to the canceled seasons of mainstream men's sports. One participant noted “*some professional leagues like NWSL and WNBA benefitted from extra coverage due to a lack of other sports*.”

Industry practitioners echoed the emphasis around the momentum of media coverage and noted the increased social media reach of women's sports in their explanation of growth. A member of this stakeholder group noted “*the momentum drew media attention/coverage and in turn drew in new fans/eyeballs*.” In addition to growth indicators, the practitioner group also elaborated on perceived reasons behind this positive trend. The athletic success of female athletes at the Olympic Games and other mega sport events were highlighted, especially in instances where men's teams lacked success, which they believed helped to drive interest, excitement, and business. Highly skilled and talented women athletes were identified as driving forces behind the momentum in women's sport. One participant noted they were “*starting to see more women's sports in mainstream media; USWNT success (especially during years where the men's team wasn't as successful on the field); Olympic years*.” The student stakeholders similarly stressed the visibility of successful women athletes and women's sport teams such as the USWNT and elaborated on the impact of their on-field success in driving media attention and visibility.

Academic stakeholders were found to echo the sentiments of previous occupation groups, capturing aspects relevant to both amateur and professional sports. This group, however, acknowledged increased participation in sport by girls and women at middle schools and high schools, outdoor adventures, and endurance events. This academic stakeholder group did not mention the growing opportunities for women to play at the collegiate or professional level. Academics identified increased media coverage and visibility as key elements that were working—their perception of this success was viewed through the lens of improved media framing and increased sponsorship support. Beyond this, academics attributed the growth of women's sports to the support and influence of high-profile male athletes, the talent levels of women athletes, and their high quality of play and success on the field. These factors were believed to play a role in the increased hype, visibility, and public popularity around women's sports.

While there was consistency in the overall sentiment of this frame, there were also nuances specific to each occupation group. Stakeholder perceptions of the growth in women's sport ultimately appear to be impacted by their occupational lens, with industry executives and practitioners focused on business aspects and athletic administrators and coaches more focused on participation, availability of sport programs, and coaching opportunities.

#### Public Support for Women's Sport and Equity

Another frame prevalent among all occupation groups was “*Public Support for Women's Sport and Equity*.” Athletic administrators, coaches, professors, students and industry practitioners all discussed the consistent efforts made and the momentum gained toward equality for women in sports as things they believed were working well. Sample comments included: “*Women in sports fighting for equality and equal pay*,” “*Boycotts/strikes /lawsuits over more equitable pay*,” and “*More teams and leagues fighting for equality through the legal system for equal pay and access to resources such as facilities, coaching, tv coverage etc. particularly with pro teams*.” In addition, the stakeholder groups recognized the solidarity among athletes and public support of women's sports as impactful change agents. For example, “*When the US soccer team supported the US women's hockey team's fight for equal pay, it created a greater feeling of solidarity*,” “P*ublic support of most pro leagues trending toward equal coverage and equal pay*,” and “*Men's sports and athletes showing support for equal pay*.” While the current state is subpar, public awareness of issues and support for change were seen as starting points and a move toward a more positive direction.

Industry executives and respondents who were unemployed at the time of the study reverberated similar sentiments, but also emphasized the greater societal movement toward gender equality in their comments. They associated public support for women's sports and solidarity among men and women athletes with the cultural shift and desire for more equitable rights within society. Sample comments to this end included “*people pushing the narrative of equal pay*,” “*more positive momentum around equity (pay, resources, etc.)*,” and “*Women have been silenced for so lon—and we're seeing a dramatic shift during COVID more than ever*.”

In addition to the two common frames discussed, unique opinions emerged within two occupation groups, namely “*Hiring of Women in Men's Sports*” and “*Women's Sports as Change Agents*.”

#### Hiring of Women in Men's Sports

Industry executives identified the recent hiring of women in men's professional sports as a positive trend in their comments. A sample quote noted, “*We were starting to see women stepping into coaching and admin roles within professional male sports leagues, like Katie Sowers with the SF 49ers*.” They discussed this as a much-needed improvement in the industry that they believed would open up doors to women beyond sports. Collectively, industry executives were focused on the business aspects of sport within the professional sport context, and they saw evolving societal views and expectations as a driving force impacting hiring practices, public support, and solidarity.

#### Women's Sports as Change Agents

**Figure 1:**
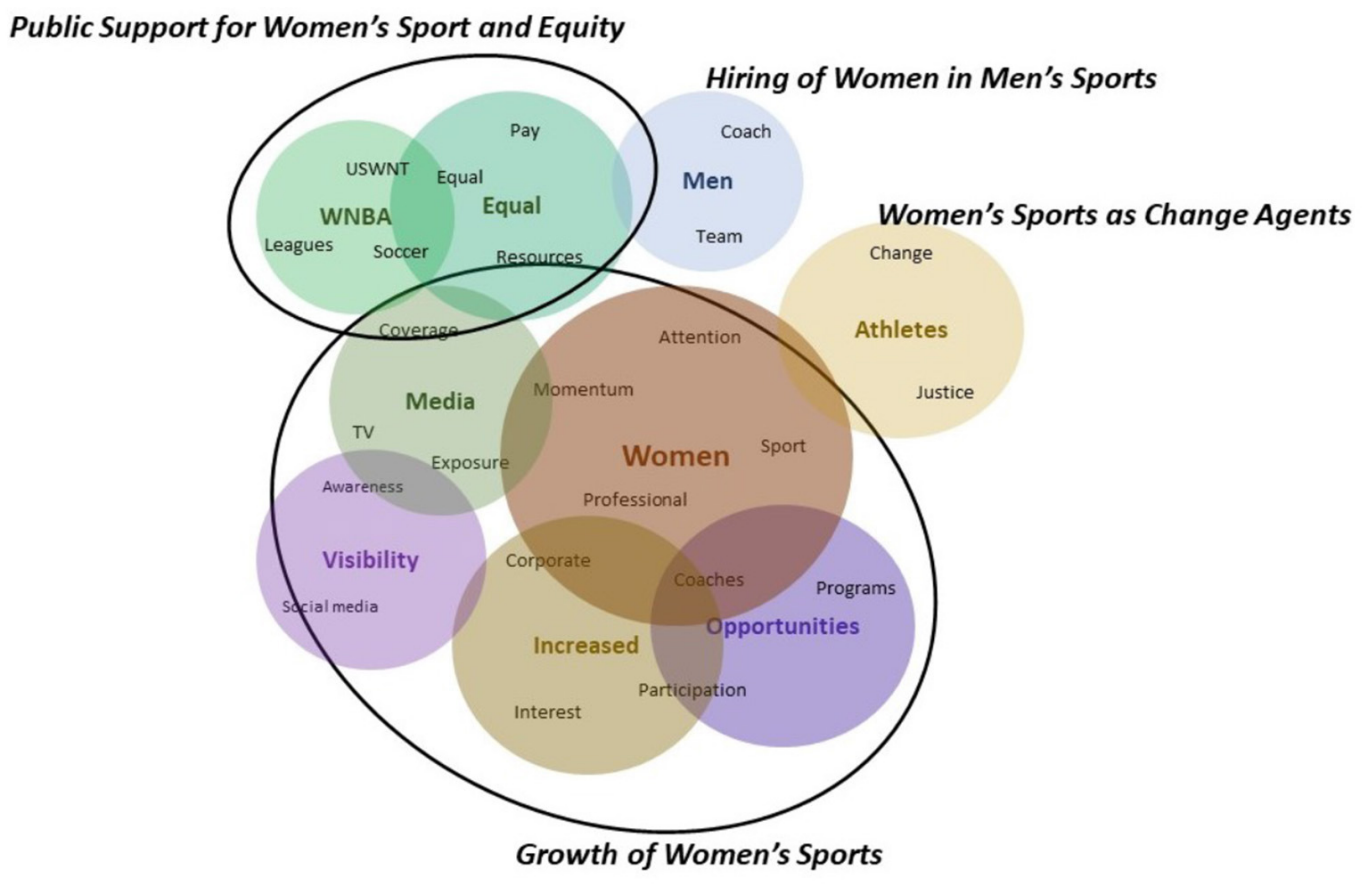
Thematic representation of the frames for question 1.

Individuals who were out of work during data collection also presented a unique perspective around what they believed was working well for women in sport and in women's sports. They recognized the leadership of women athletes and women's sports in driving the social justice movement and contributing to social change. Sample quotes read as “*Women's sports seem to incorporate issues of equality and social justice to a greater degree in the foundation of their leagues and organizations*” and “*athletes are using their own platform to bring about social change*.” While women athletes have been very involved in raising awareness on social justice issues and serving as leaders in their communities, this emerged as a unique perspective within the positive trends in women's sports pre-pandemic within this stakeholder group. Please see Figure 1 for the thematic representation of the frames discussed.

#### Perspectives by Age Cohort

Similar to the perceptions of occupation groups, our examination of generational perspectives revealed some consistency among respondents in their responses to what has been working well in women's sports. All age cohorts perceived the growth of women's sports as a positive development in the industry along with girls' and women's increased participation in sport. Generation Z and Millennial respondents tended to focus their comments around gender equality and the influence of women athletes in driving change. Generation Z discussed gender equality in sports heavily, especially at the collegiate level, focusing on equal opportunities to play, availability of better resources, and athletes and coaches advocating for their sports. A sample comment included “*The continued progress of fighting for equality (even though there's still a long way to go) and amplifying the voices of female athletes and coaches*.” Millennials emphasized the influence of athletes in raising the popularity of women's sports, raising awareness of the issues surrounding women's sports such as equal pay. Some comments were “*Some names were starting to become really well-known around the world, esp. in soccer, basketball and, in Europe, handball*,” “*continued rise of female athlete voice on a more mainstream level” “More female athletes speaking out and fighting for equality*.”

### Frames Within “Challenges”

Consistency was noted across stakeholder occupation groups relative to anticipated challenges in women's sports post-pandemic. With some nuances, groups generally agreed that the potential decline or loss of hard-earned resources could be problematic, with many fearing an interruption to the momentum women's sport had developed prior to the COVID-19 pandemic.

#### Maintaining Momentum

As previously discussed, “*Growth of Women's Sports”* was identified as a positive trend across all occupation groups. In coherence, “*Maintaining Momentum*” was brought to the fore by all occupation groups as a key obstacle for women's sports post-pandemic.

Athletic directors and coaches discussed the potential impact of declining resources and voiced fears around the possible elimination of women's programs and/or coaching positions/staff. Sample comments illuminate this noting “*Decreased revenue for programs mean funds tend to be funneled to the men's programs with the explanation of COVID as an alibi*” and “*The ability to function with excellent coaches and have the funds to continue to run will be a challenge*.”

For industry executives, practitioners and respondents who were unemployed, anticipated challenges were related to the business impacts and bottom line of women's sports. The disappearance of live attendance and its impact on game day revenues, sponsorship, and media rights were discussed in relation to the sustainability of women's sport leagues, accessibility, and the need for consumer support. Sample quotes include “*TV revenue remains, corporate sponsorship has declined, and so has merch/F*&*B, revenue dollars are a major concern*,” “*Governing bodies are facing major financial burdens with limited access to revenue via broadcasting, ticket sales, merchandise etc*.” and “*Huge $$$ investment in resources for female athletes* & *their respective leagues are needed to continue with game play*.” Furthermore, these groups discussed the continuation of media attention and momentum built prior to the pandemic as a perceived challenge noting, “*Male sports will push to capture programming and look for ways to make up for the lost revenues, which may take funds away from women's sports*.”

The perspectives of academic respondents aligned with stakeholders from collegiate sports and professional sports. They also shared concerns around the loss of women's sports programs due to budget cuts and women's sports ability to maintain media attention, visibility and corporate support at the professional level. Sample comments read “*With the state budget cuts, youth sports and collegiate programs might be cut, opportunities to play may decline for women and girls*” and “*Maintaining the momentum women's pro sports had prior to pandemic might be a concern. Media attention, visibility, sponsor support/investment, fan attendance, resources, interruption to international mega events*.” Student stakeholders echoed their professors' perspectives, elaborating on potential challenges in both the collegiate and professional sport contexts.

The various occupation groups identified unique challenges relevant to their day-to-day experience. Athletic administrators and coaches, for example, foresaw the status quo persisting and the sports industry remaining a gendered institution favoring men, while industry executives discussed corporate support as “All Talk.”

#### Persistence of Status Quo

The second frame emerging from the comments of athletic administrators and coaches was “*Persistence of Status Quo*.” Prior to the pandemic, although some improvements were taking place, the gendered institutional structure within the sports industry was still dominant. Athletic administrators foresaw a continuum of status quo through old-school hiring patterns, a lack of opportunities provided to women and people of color, and hiring practices along gendered lines. A sample comment was “*Until the NCAA, government, and University presidents require change, athletics will continue to be modern day slavery in which white males and white women benefit from the labor of minorities, majority Black students and staff while keeping them in poverty*.” For coaches, “*Persistence of Status Quo”* was related to a lack of Title IX compliance, limited pay persisting for women coaches and administrators, and challenges in preserving non-revenue programs. Some comments reflecting these ideas were “*Many colleges and universities continue to run athletic departments that are not Title IX Compliant—and NCAA seems to look the other way*” and “*Many coaching positions already do not pay enough, this will continue to be an issue and impact female coaches*.”

#### All Talk

**Figure 2:**
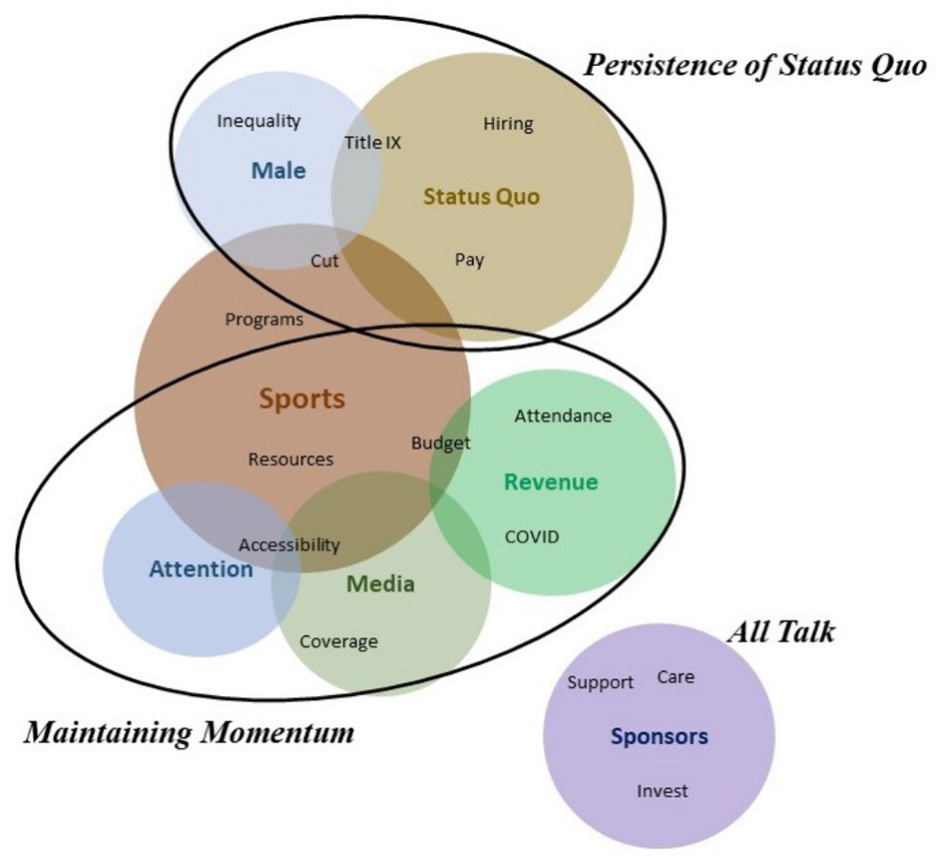
Thematic representation of the frames for question 2.

This frame refers to the perceived issue of corporations' voicing verbal commitment to gender equality and women's sports but failing to follow through with meaningful actions like financial or in-kind support. Representative quotes note “*we also need people, companies, brands etc. to action their words*,” “*Everyone says they care about women and they care about equality. But are you watching the games? Are you supporting the growth of these leagues? Are you opening your wallets and using your voice to propel women in sports?*,” and “*How men in positions of power act not matching what they say*.” Industry executives expressed deep frustration with the limited actions taken by potential corporate sponsors. Please see Figure 2 for the thematic representation of the frames covered.

#### Perspectives by Age Cohort

Analysis of responses to challenges anticipated post-pandemic showed coherence in generational perspectives. Baby-boomers, Generation X, Millennials and Generation Z all expressed deep concern around the interruption of momentum in women's sports as a result of the forced stoppages caused by the pandemic.

### Frames Within “One Change to Make”

For the final question, respondents were asked to identify the change they believed would have the greatest impact on women's sports. All occupation groups identified greater media coverage as an essential change required in women's sports. Beyond this, improved marketing approaches, male advocacy, improved representation, and collegiate athletic reform were highlighted by the various occupation groups.

#### Greater Media Coverage

All occupation groups identified “*Greater Media Coverage”* as an essential driver critical for change in women's sports. Respondents recognized the power of the media and saw media coverage as a centerpiece that could be used to propel improvements around public awareness while enhancing broader appeal for women's sports, sponsor interest, and ultimately higher revenues. Greater media coverage, especially on mainstream media outlets during primetime programming, was viewed as a precursor to sustainable women's sports and key to better overall business outcomes. Respondents noted “*Women's sports need more mainstream media support*” and “*Increased visibility and promotion drive awareness and get fans to tune in. This needs to come from the major sports networks*.”

Beyond enhanced programming and coverage, the content of media stories, proper framing, and engaging programming were also acknowledged. A representative comment read “*Creating engaging behind the scenes programming of the journey, trials and tribulations of our women athletes—giving current and prospective fans a better connection to the players, teams and leagues*.”

#### Better Marketing

Industry executives, respondents who were out of work, and academics all noted “*Better Marketing”* as a key element for women's sports and athletes moving forward. These occupation groups suggested complementing increased media coverage with better marketing and branding strategy to develop emotional connections with consumers. Comments to this end recommended “*More storytelling about the athletes to connect with fans*” and “*Develop the athletes off of the court/field—the better business-minded they are about their own brand, the more opportunity for revenue they will have individually*.”

#### Male Advocacy

Industry executives and academics also discussed male allies and advocates as an instrumental factor for change. They identified male athletes as influencers and highlighted their potential to drive awareness around women's sport, improve equal pay and work conditions, and expand women's sport fan bases. Sample comments explained that “*Male athletes supporting and advocating for women's sport will be a catalyst that leads to male fans supporting*” and “*If people see the most talented male athletes talking about their female counterparts and advocating for equal pay, I'd hope that it would have a ripple effect*.”

#### Can't See Can't Be

Athletic administrators, coaches and students all mentioned the importance of increased representation of women in leadership positions. For these occupational groups, having women in leadership positions was not only about visibility and gender equity, but also about having the power to make decisions and influence change. They suggested “*policies and quotas at the governance level that support gender equity in administration and leadership, women need genuine decision-making power in sport organizations*” and “*You can't be what you can't see, so this is the time and opportunity in 2020 to unite, have a shared voice and to lead the changes we all want to see*.”

#### Collegiate Athletic Reform

The last frame identified was “*Collegiate Athletic Reform”* and it emerged within the comments of coaches and academics. Within this frame, respondents discussed two separate aspects of collegiate athletics. The first theme focused on gender equity and the need for a formal reform of NCAA mandates that hold universities more accountable for their actions. Sample comments included “*Equal opportunity and facilities at all colleges/universities enforced by NCAA pressure*,” “*Progressive policy changes, structural changes especially now that seasons are canceled”* and “*There needs to be collegiate athletics reform legislation that stops lavish expenditures on facilities and puts caps on coaches' salaries*.”

**Figure 3:**
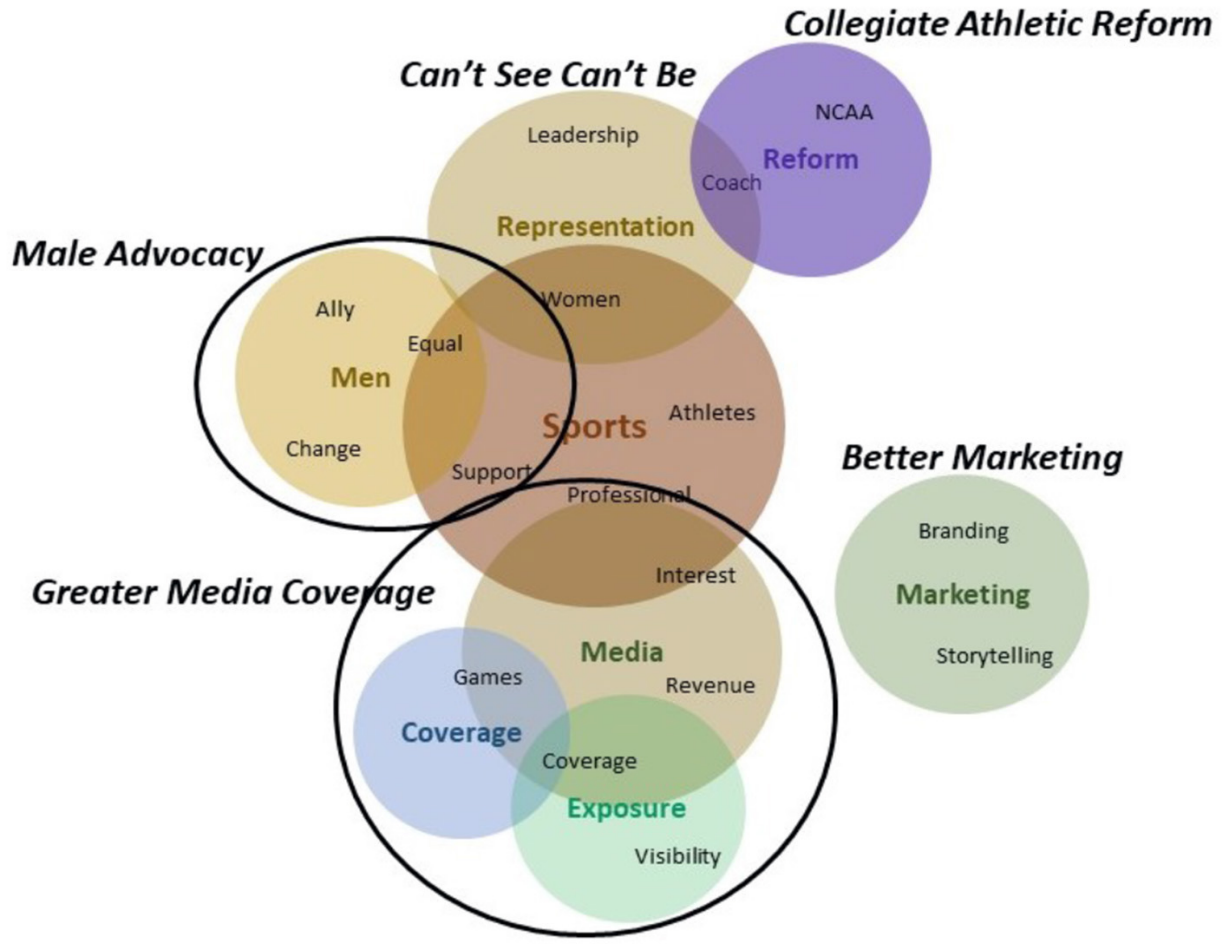
Thematic representation of the frames for question 3.

The second frame was devoted to the needs and rights of student-athletes, especially their right to have medical coverage and a safe environment, free from abuse. Sample comments called for “*a reform that mandates significant expenditure increases on student-athlete health and protection—full coverage of medical expenses, athletic injury insurance, long term disability and a trust fund to cover the anticipated long-term costs of dementia, ALS, CTE for athletics*” and “*reinforcement of regulations and by-laws to keep student-athletes safe from predators, abuse*.” Please see Figure 3 for the thematic representation of the frames uncovered from respondent comments to key change to improve women's sports.

#### Perspectives by Age Cohort

Our generational analysis for this question revealed a new frame based upon the comments of Generation X participants. “*Improved Governance of Sport*” was a common theme among this cohort with a focus on the need to improve the governance of Olympic sports and develop and enforce policies to establish gender equity and better funding. Participants advocated for “*Policies and quotas at the governance level that support gender equity in administration and leadership, women need genuine decision making power in sport organizations*,” “*funding and public support for funding toward women's sport—especially at the national sporting body level where there isn't transparency as to the % of national sports spending on women's vs. men's sport*,” “*support of non-broadcast Olympic sports, remove federated model from sport, force diversification across leadership*.” In addition to this new frame, age cohorts suggested increased media coverage and better framing as key changes to improve the status of women's sports.

## Discussion

This research was inspired by an academic problem—the issue wherein so little of our research on women's sport seems to break through to inform key stakeholders or affect change across the larger women's sport landscape. The purpose of our research was therefore to apply a problem solving framework to evaluate the current women's sport landscape through the perspective of a variety of women's sport stakeholder groups in order to enhance our understanding of the larger women's sport ecosystem and inform the re-framing of our research agendas. Our findings suggest while similarities across stakeholder groups exist in terms of perceptions of the women's sport space, one's occupational lens and age impacts how individuals frame problems and set goals, creating siloed differences in perspectives. While it is not novel to suggest that we frame problems in different ways based upon our circumstances or that we establish different goals accordingly, it is imperative for stakeholders in women's sport to understand that our respective occupational strengths are necessary to fully realize our individual occupational goals. Perhaps more importantly, our collective goal of advancing gender equity in sport cannot be solved by any one individual, team, sector, or organization. We are stronger and better resourced problem solvers as a collective force.

Giles (2008) spoke to the value of broadening scholarship to include practitioner voices as co-generators of knowledge. But how do we move forward with this notion in mind? What needs to be done to close the re-framing loop and turn ideas into action? Among the various stakeholder groups represented through this work, we found broad acceptance of the notion that the growth and public support of women's sport are viewed as key markers of success, a decline in resources due to the impacts of the pandemic was a shared concern, and enhanced media coverage was collectively deemed an essential necessity to drive change. Beyond this, however, we found several strategy differences, underscoring the need to improve communications between stakeholder groups to better understand the many narratives of practice. Moreover, a need to gain the perspectives of outsiders was made evident through stakeholder views that championed the role of male allyship and athlete influencers as vehicles for change. Wedell-Wedellsborg (2020) refers to these types of “outsiders” as “boundary spanners” and defines them as people who understand but are not fully part of your world (p. 156). The observations of boundary spanners function to stimulate new ways of thinking. When dealing with a complex problem like gender equity in sport, outsiders' perspectives can be a powerful shortcut to identifying new framings and may offer researchers a more complete understanding of the current sport landscape (Wedell-Wedellsborg, 2020).

The ultimate integration of multiple perspectives into our research strategy has the potential to provide for a more interactive approach to our work and could help overcome the identified problem of siloed thinking (Wedell-Wedellsborg, 2020). It is important to note the point of the reframing process is not to arrive at one final conclusion—rather it is to move us into discovery-oriented conversations that embrace a learning mindset to better position us as problem solvers in order to move toward more creative solutions (Wedell-Wedellsborg, 2020). The key tactical challenge of course, is knowing which frames to focus on and prioritize in our exploration. Wedell-Wedellsborg (2020) suggests organizing frames into three categories: those that are surprising, simple, and significant if true.

Surprising frames are defined as those that break with a mental model that problem owners may have grown accustomed to. Within our findings, perhaps the most surprising outcome was not a specific frame, but rather the combined variety of frames and the more granular nuances that shaped various stakeholder perceptions. Alongside this, the identified disconnect between academics and practitioners was eye-opening. While we were united around the belief that the current growth of women's sports and the broader public support of the women's sports movement were hallmarks of success prior to the pandemic, the antecedents behind these beliefs appear to be marked by important variance. Industry executives, among the key decision makers in women's sports, viewed growth in terms of increased public awareness, better corporate support, and enhanced media coverage. They also highlighted the importance of women being hired in men's professional sports and emphasized the growing reach of social media in the women's sport space which reinforces the motivational divide that exists between stakeholder groups in women's sport. Sport practitioners are focused on business imperatives and are looking for bite-sized research insights that can be readily digested and leveraged to impact their bottom line *today*. Comparatively, academics are not bound by the same industry demands, and thus have greater flexibility to consider the longer-term goal of sustainable change in the women's sport space. As we re-frame our research agenda, we must ask ourselves how much of our current research is directly devoted to practitioner issues? What “sticky stats” might we be able to develop through our research to help our industry colleagues negotiate better corporate investment? Are there opportunities to reposition our research with different framings for different audiences? If we are to truly embrace a “learning mindset,” these are all important questions to consider, particularly given our findings are representative of individuals who are all highly invested in the women's sport realm. Existing variance among these stakeholder groups alone underscores the impact of functional fixedness and highlights the range of factors and influences we are missing in our research.

The second organizational strategy recommended by Wedell-Wedellsborg (2020) is the simple frame, meaning straightforward solutions wherein a small change in behavior could potentially have a profound, far-reaching impact. In light of our findings, it appears the simplest solution at our disposal as academics may be to ensure that our research findings are appropriately framed for different audiences and *accessible* to audiences outside of academia. In short, we need to improve our messaging and consider how we engage with industry to help them better understand the benefits of our academic lens. The value proposition for industry to engage with academia is more than undergraduate projects and internship opportunities. Academics are professionals trained to think critically and we have the knowledge to apply deep contextual expertise. Strategic academic partnerships could offer industry colleagues data, metrics and strategy grounded in academic rigor and in addition, provide rich insights that help drive revenue and innovative business decisions. In the case of women's sport, a collaboration of stakeholders who embraced data sharing could help to fill the data void that exists across the women's sport market with research thoughtfully tailored to craft the best possible business case for women's sport. The uniqueness of this rigorous brand of knowledge could serve to keep industry top of mind and simultaneously support our academic goal of sustainable change. Communication, however, is required to educate industry colleagues of the value of collaborations, develop realistic goals, define respective expectations, and ensure appropriate attribution.

Finally, Wedell-Wedellsborg (2020) recommends testing framings that challenge our assumptions and beliefs about problems with a focus on frames we may *not* believe in. When re-framing, he notes problem solvers must be careful about trusting their intuition given “intuition is built from your past” and creative solutions often involve “transcending past experiences” (p. 147). To this end, we focus on the generational divide that emerged from our findings. Millennial and Gen Z respondents were more attuned to the role of women athletes driving change and bringing increased awareness to women's sports. Their responses reflected the observations of women's sports scholars who point to the importance of athletes' voices on digital platforms (Bruce, 2016; Pegoraro et al., 2019). They also starkly contrasted with Gen X participants who viewed change from a more traditional lens, calling on policy reform to affect change (e.g., Sabo et al., 2016; IOC, 2018). Gen X respondents, by virtue of their age, are more likely to find themselves in decision making roles. Their experiences are also more likely to be groomed by the impact of Title IX and rooted in the idea that women's sports are a moral imperative. Next-generation respondents, however, have been shaped by different cultural contexts and their experiences may be more attuned with the changing model of sport delivery. It is noteworthy that their perspective also appears to be better aligned with the industry focus of building the business case for women's sport through data-driven insights. In reality, the overarching challenges facing women's sports likely require thoughtful solutions that are informed by both of these perspectives. It is not an either/or situation, but rather a both/and scenario. Re-framing research questions that are informed by our past, but open to cultural immediacy have the potential to provide us with both new perspectives and more impactful solutions.

### Limitations

As with all research endeavors, our work is not without its limitations. First, while the COVID-19 global pandemic enforced an unprecedented global sports stoppage which provided a unique opportunity for this research, it may have also impacted how respondents answered the questions. Second, using a purposive sampling to collect insights from sport industry professionals and academics alike may have also provided a narrow response sample. The respondent demographic profile was not reflective of intersectional identities, as it was predominantly Caucasian, dominated by respondents who identified as female, and had limited representation from Generation Z. Lastly, the respondents were all knowledgeable of women's sport, which was the goal, but this may have also provided narrowed response data while including those less knowledgeable may have provided some additional data to help re-frame the issues.

### Conclusion

This research provided insight into what key stakeholders in women's sport thought was working, what the perceived challenges were, and where they believe efforts need to be focused for women's sport to continue to grow. Through the analysis of responses, and the use of a reframing process, we have provided examples of where stakeholders can come together—opportunities where we could begin to think differently. Previous work has suggested underlying differences in functional background, education, and personality can be positively related to performance, particularly as they contribute to the facilitation of creativity and group problem solving (Mannix and Neale, 2005). Moreover, the asset of diversity has been found to help bridge understandings of the marketplace and enhance the power of minorities (Mannix and Neale, 2005). This work points to a similar conclusion. We believe the possibilities are endless if we push ourselves, and others, toward “rethinking and unlearning,” a critical cognitive skill set in our increasingly turbulent world (Grant, 2021, p. 2). This means getting out of our own way and thinking beyond traditional structures. This means letting go of past assumptions and making room for multiple solutions at multiple levels. It means working together for the common good in women's sport. We do not need a monolithic statistic or policy but given the dearth of knowledge specific to women's sport, there is no time for us as key stakeholders to be repetitive in our work or competitive with one another. It is inefficient for us to be working on similar problems in individual siloes, developing solutions that lack the wisdom of crowds or applicability to the stakeholders we are trying to serve. Time is of the essence. We are strongest together when all stakeholders have the chance to apply their individual strengths, whether it be through occupational insights, generational insights, or data sharing. As stakeholders of women's sport, we are not serving each other to the highest potential until we are able to make this happen! It is time for us all to find comfort in the discomfort of change and as we rethink our research agendas as academics, we have an opportunity to multiply our force by reaching across the aisle to our fellow stakeholders to better achieve our individual and collective goals—bringing gender equity in sport closer to fruition.

## Data Availability Statement

The original contributions presented in the study are included in the article/supplementary material, further inquiries can be directed to the corresponding author/s.

## Ethics Statement

The studies involving human participants were reviewed and approved by University of Minnesota Institutional Review Board. Written informed consent for participation was not required for this study in accordance with the national legislation and the institutional requirements.

## Author Contributions

All authors listed have made a substantial, direct and intellectual contribution to the work, and approved it for publication.

## Conflict of Interest

The authors declare that the research was conducted in the absence of any commercial or financial relationships that could be construed as a potential conflict of interest.

## Publisher's Note

All claims expressed in this article are solely those of the authors and do not necessarily represent those of their affiliated organizations, or those of the publisher, the editors and the reviewers. Any product that may be evaluated in this article, or claim that may be made by its manufacturer, is not guaranteed or endorsed by the publisher.

## Footnotes

^1^Tiel, B. (2002). *The Underrepresentation of Female Administrators in Intercollegiate Athletics*. Tiffin, OH: Tiffin University (Unpublished manuscript).
